# Efanesoctocog Alfa for the Management of a Life-Threatening Intracranial Haemorrhage in Mild Haemophilia A: A Case Report

**DOI:** 10.7759/cureus.118716

**Published:** 2026-10-28

**Authors:** Laura C Seoane, Paula S Fuentes, Patricia S Diaz, Manuel R Lopez

**Affiliations:** 1 Hematology and Hemotherapy, Complejo Hospitalario Universitario de Pontevedra, Pontevedra, ESP; 2 Hematology and Hemotherapy, Hospital Universitario Álvaro Cunqueiro, Instituto de Investigación Sanitaria Galicia Sur (IISGS), Estructura Organizativa de Xestión Integrada (EOXI) de Vigo, Vigo, ESP

**Keywords:** chromogenic assay, efanesoctocog alfa, factor viii, intracranial haemorrhage, mild haemophilia a, perioperative management

## Abstract

Mild haemophilia A (HA; baseline factor VIII (FVIII) 5-40 IU/dL) is usually free of spontaneous bleeding, but high-energy trauma or major surgery can precipitate life-threatening haemorrhage requiring prolonged haemostatic coverage. These patients are typically treated on demand, are rarely trained in self-infusion, and lack an established treatment model. Efanesoctocog alfa, a first-in-class FVIII replacement therapy with a high sustained activity (HSA), has been studied almost exclusively in severe HA. We report a 33-year-old man with mild HA (baseline FVIII 22 IU/dL) who sustained a severe traumatic left temporal epidural haematoma with associated skull-base and pelvic fractures. After an initial standard FVIII bolus, he was managed with efanesoctocog alfa using a progressively spaced regimen, with FVIII monitored by chromogenic assay corrected per the manufacturer's recommendation. Complete radiological resolution occurred by week 8 without rebleeding; no inhibitor developed. The bleed was controlled with only seven infusions in the first 14 days and without central venous access, tapering to conventional weekly dosing. We discuss why the pharmacokinetic profile of efanesoctocog alfa is particularly advantageous in the mild phenotype and how the same properties extend to major surgery, together with a cautious appraisal of its cost profile, which is strongly dependent on the healthcare system.

## Introduction

The label “mild” describes the everyday life of patients with mild haemophilia A (HA) well - no spontaneous bleeding, often no prophylaxis, and no experience with self-infusion - but it ceases to be reassuring when high-energy trauma or major surgery occurs. The clinician then faces an uncomfortable paradox: a treatment-naïve patient who suddenly requires sustained high haemostatic levels for weeks. Efanesoctocog alfa (Altuviiio/Altuvoct; Sanofi, Cambridge, MA, USA, and Sobi, Stockholm, Sweden) is a first-in-class factor VIII (FVIII) replacement therapy that decouples recombinant FVIII from endogenous von Willebrand factor to overcome the half-life ceiling, achieving a terminal half-life roughly three to four times that of conventional FVIII products [1,2]. Clinically, once-weekly prophylaxis at 50 IU/kg in previously treated patients with severe HA achieved a mean annualised bleeding rate of 0.71 (95% CI, 0.52-0.97), superior to prestudy FVIII prophylaxis, maintained mean FVIII activity above 40 IU/dL for most of the dosing interval, and resolved 97% of bleeding episodes with a single injection [1]; comparable efficacy and safety were reported in children [3], and a pooled perioperative analysis showed that major surgery could be managed with substantially lower factor consumption than with an extended-half-life comparator [4]. This evidence, however, derives almost entirely from severe HA, since patients with mild and moderate disease were excluded from the pivotal trials [1,3]; experience in the mild phenotype is confined to isolated real-world reports [5]. We present a patient with mild HA who sustained a traumatic epidural haematoma that was managed conservatively, without surgical evacuation, and in whom efanesoctocog alfa was used to sustain haemostatic cover throughout the reabsorption process.

## Case presentation

A 33-year-old man with mild HA (baseline FVIII 22 IU/dL; F8 c.1372C>T, p.Arg458Cys in hemizygosity), previously managed on demand and responsive to desmopressin, with no prior FVIII self-administration, sustained a severe traumatic brain injury with a left temporal epidural haematoma (17 mL on 24-hour computed tomography), a complex petrous bone fracture, and a stable pelvic fracture (Figure 1). He was neurologically intact on admission (Glasgow Coma Scale 15, no focal deficit) and haemodynamically stable. The neurosurgical team elected conservative management, without surgical evacuation of the haematoma.

**Figure 1:**
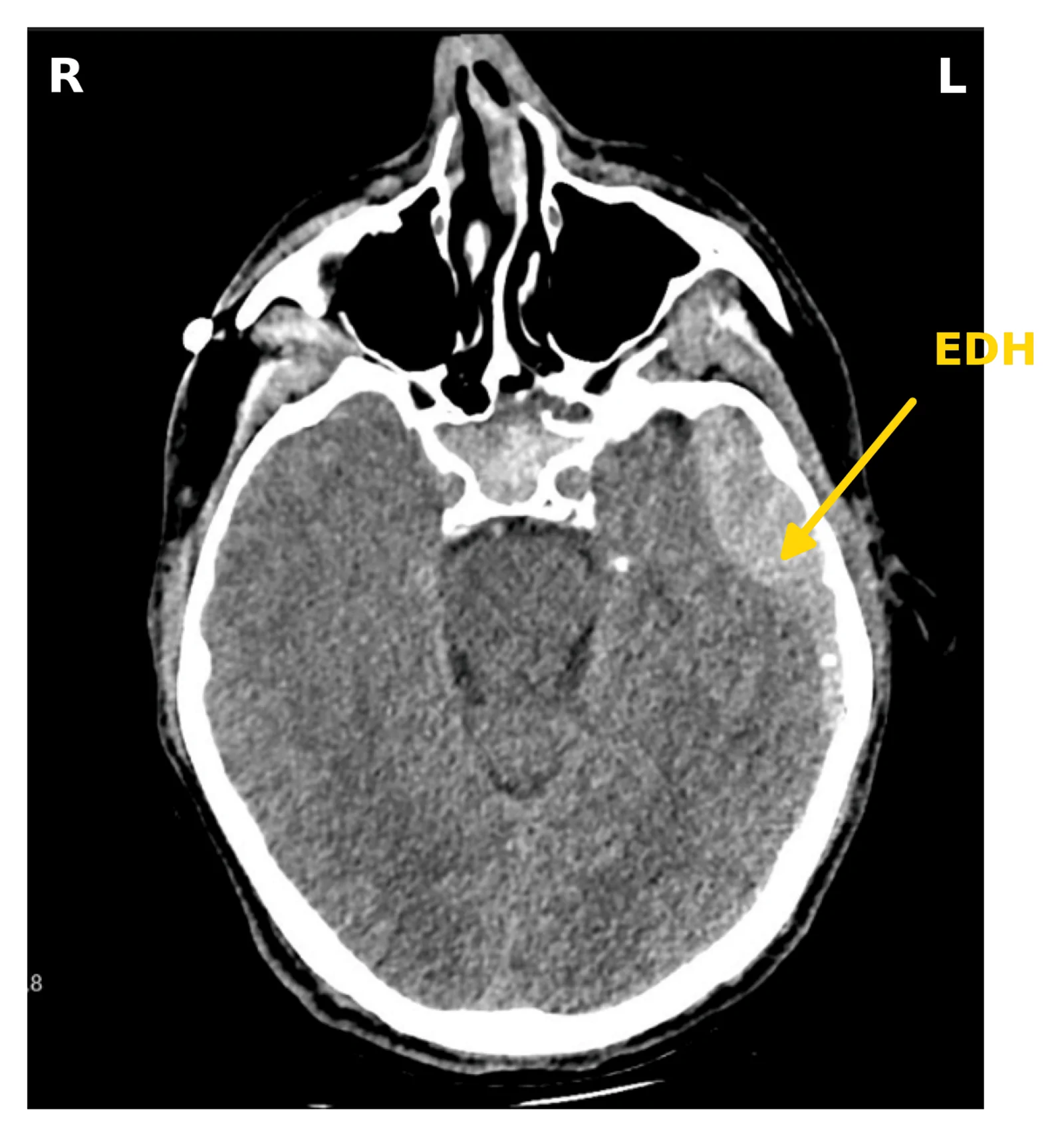
Axial non-contrast CT at presentation Axial non-contrast CT at presentation showing a left temporal epidural haematoma (arrow; approximately 2 cm thick; patient's left corresponds to the right of the image, radiological convention) with an associated left temporal bone and petrous fracture, ossicular chain subluxation, and left temporal pneumocephalus. R: right; L: left; EDH: epidural haematoma

After an initial standard FVIII bolus (50 IU/kg) at the referring centre, efanesoctocog alfa was started 12 hours later as induction at 25 IU/kg every 24 hours. Guided by trough FVIII levels, the dose was increased to 40 IU/kg every 48 hours on day 3 (trough 51 IU/dL) and then reduced to 30 IU/kg every 48 hours from day 5 (trough 80 IU/dL), which was maintained through day 21 (trough 81 IU/dL on day 7). Because the recommended one-stage clotting reagent (Actin FSL; Siemens Healthineers, Erlangen, Germany) was unavailable, FVIII activity was monitored with a chromogenic assay (Electrachrome®; Werfen, Barcelona, Spain), dividing the result by 2.5 as recommended in the manufacturer's summary of product characteristics to correct for assay overestimation [6]. During this fixed 30 IU/kg every-48-hour schedule (days 5-21), activity was stable and predictable, with peaks of approximately 130 IU/dL and troughs of approximately 70 IU/dL. Across the intensive period, these values reflected the accumulation expected from the ultra-long half-life and remained comfortably within the World Federation of Haemophilia (WFH) targets for the acute and subacute phases of intracranial haemorrhage (Figure 2 and Table 1) [7]. Beyond day 21, as radiological improvement was confirmed, the regimen was progressively spaced to 2,500 IU every 96 hours and then to 3,000 IU weekly in an ambulatory setting, maintaining levels consistently above target (>30-50 IU/dL) (Figure 3).

**Figure 2:**
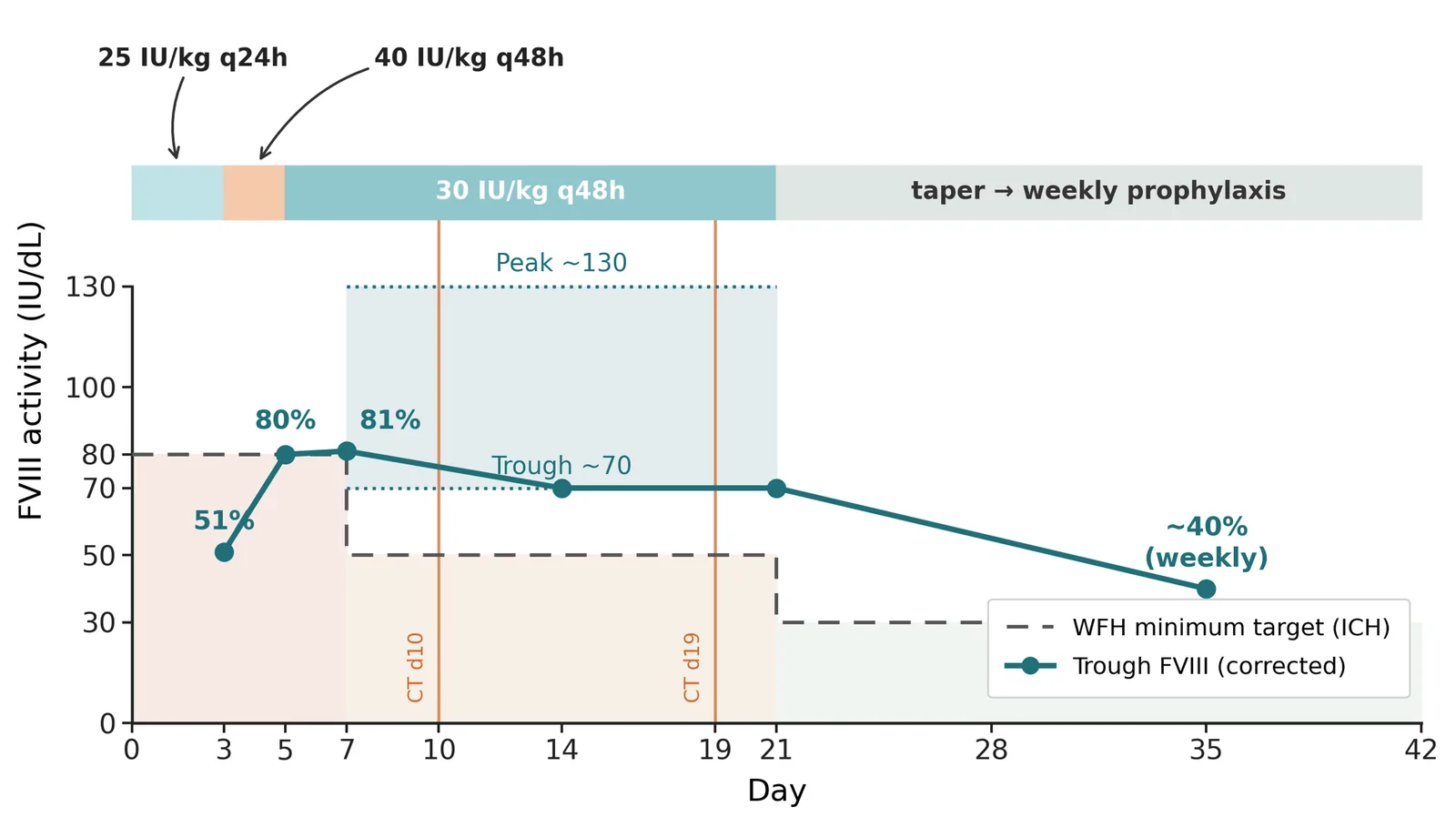
Corrected FVIII activity over time (chromogenic assay, result divided by 2.5) Measured trough levels (teal) rose from 51 IU/dL on day 3 to approximately 80 IU/dL on days 5-7; during fixed 30 IU/kg dosing every 48 hours (days 5-21), activity oscillated between a trough of about 70 and a peak of about 130 IU/dL (shaded band). The dashed line marks the WFH minimum targets for intracranial haemorrhage (ICH) (>80% on days 0-7, >50% on days 8-21, >30% thereafter). Dose phases are indicated along the top; orange lines mark the follow-up CT scans on days 10 and 19. WFH: World Federation of Haemophilia The figure was created by the authors using Python (Matplotlib), with the plotting code developed with the assistance of a generative artificial intelligence tool, Claude (Anthropic, San Francisco, CA, USA).

**Table 1:** Factor VIII activity (FVIII:C) during treatment FVIII activity was measured by chromogenic assay (Electrachrome, Werfen, Barcelona, Spain), with the result divided by 2.5 as recommended in the efanesoctocog alfa (Altuvoct) summary of product characteristics. The laboratory reference range for FVIII:C was 50-200 IU/dL for all determinations. Treatment day 1 corresponds to the start of efanesoctocog alfa. An FVIII inhibitor (Bethesda assay) was negative (<0.6 BU/mL) one month after discontinuation. FVIII:C: factor VIII coagulant activity; BU: Bethesda units; q24h/q48h: every 24/48 hours

Treatment day	Regimen	FVIII:C, IU/dL
Baseline (pre-treatment)	On demand	22
Day 3 (trough)	25 IU/kg q24h → 40 IU/kg q48h	51
Day 5 (trough)	40 → 30 IU/kg q48h	80
Days 7-21	30 IU/kg q48h	81 (day 7); 70 trough/130 peak
Days 21-44	30 IU/kg twice weekly	60 trough/120 peak
Days 44-132	50 IU/kg weekly	40 trough/90 (day 3-4)/150 peak
One month after discontinuation	None	26

**Figure 3:**
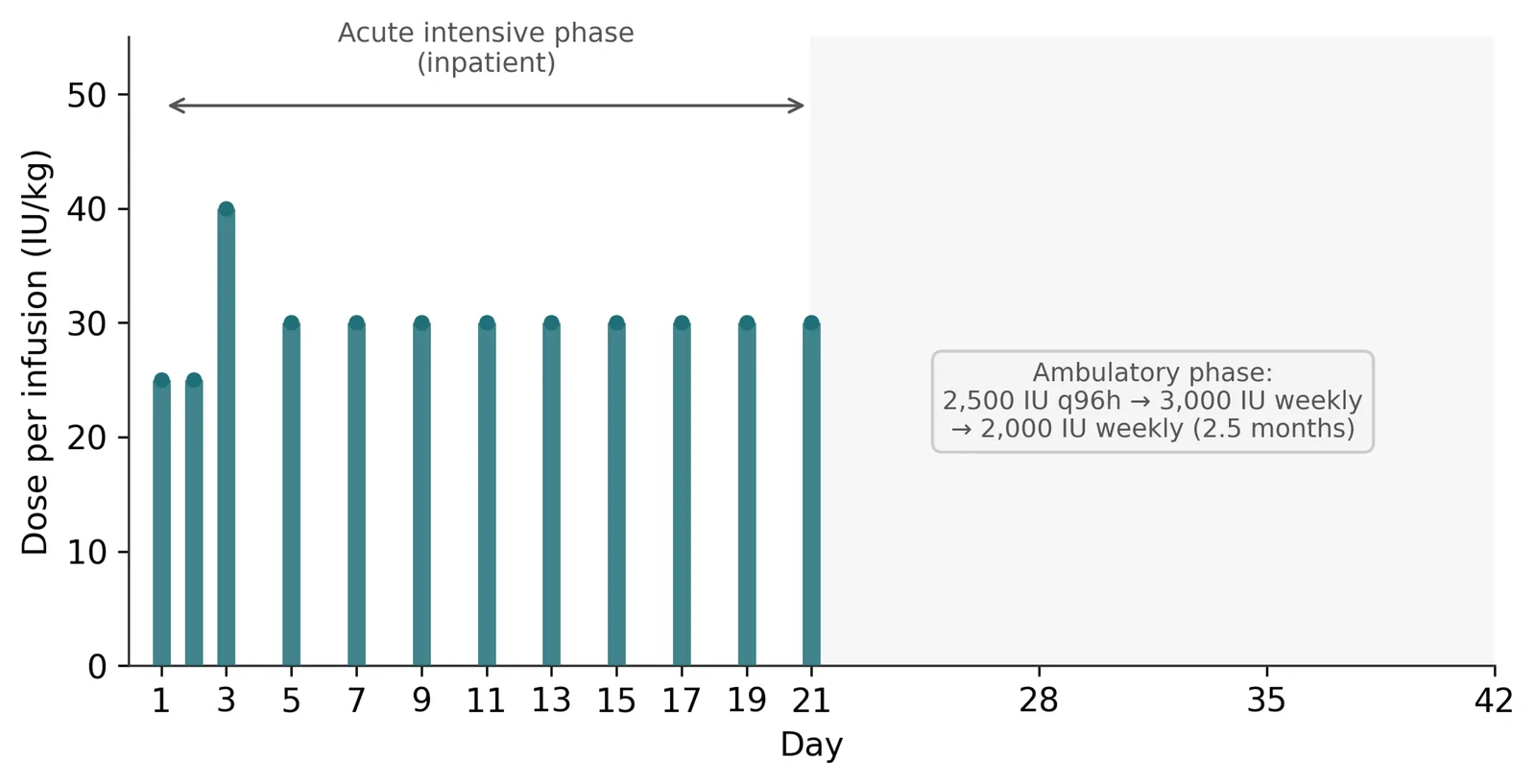
Efanesoctocog alfa dosing schedule Each vertical bar represents one infusion (height = dose in IU/kg). After induction (25 IU/kg) and a brief 40 IU/kg step guided by trough levels, the patient received 30 IU/kg every 48 hours through day 21, followed by an ambulatory phase progressively spaced to weekly prophylaxis. The intracranial bleed was controlled with a low number of infusions relative to standard-half-life concentrates. The figure was created by the authors using Python (Matplotlib), with the plotting code developed with the assistance of a generative artificial intelligence tool, Claude (Anthropic, San Francisco, CA, USA).

Complete radiological resolution of the haematoma was confirmed at week 8, with no rebleeding. Weekly prophylaxis was deliberately maintained beyond radiological resolution - for a further three months, tapered to 2,000 IU/week before discontinuation. This decision was based on three considerations: intracranial haemorrhage carries a substantial risk of rebleeding during the reabsorption and healing phase, well after imaging normalises [7,8]; the patient had concomitant petrous and pelvic fractures still consolidating; and continued haemostatic cover was considered prudent during mobilisation, rehabilitation, and neurosurgical follow-up in a treatment-naïve patient with no prior prophylaxis experience. Baseline FVIII one month after discontinuation was 26 IU/dL, confirming the absence of an inhibitor. All ambulatory infusions were administered in the day-hospital setting, with no central venous access device required.

## Discussion

Two features are particularly relevant to the mild phenotype. First, the number of infusions was markedly low - nine in the first 14 days (one standard-half-life bolus plus eight doses of efanesoctocog alfa), versus the 14-28 theoretically required with standard-half-life concentrates. In a patient who had never self-infused, this meant fewer venepunctures, fewer hospital visits, and, critically, avoidance of a central venous catheter in a polytrauma patient with compromised venous access and its potential adverse events (infection, disconnection, thrombosis). Second, a point that requires nuance: efanesoctocog alfa reduces the number of exposure days but prolongs the time FVIII remains in circulation, so immunological exposure is not straightforwardly reduced. Although intensive treatment is a recognised trigger of inhibitor development in non-severe HA [9], we are therefore cautious in extrapolating a protective effect; no inhibitor developed here, but the net effect of ultra-long-acting molecules on this risk remains to be defined. Monitoring deserves its own comment: with this agent, omitting the manufacturer-recommended chromogenic correction overestimates activity and may lead to excessive dose spacing - a practical pitfall that is far from trivial during a life-threatening bleed.

One question deserves an explicit answer: why the haematoma was not surgically evacuated. The decision was made by the neurosurgical team and was driven by the bleeding disorder, with a conservative approach preferred over an operation judged to carry an unfavourable risk balance in a patient with haemophilia. Stating this plainly matters because it reframes what the drug contributed. Efanesoctocog alfa did not spare an operation that was otherwise indicated; it made the conservative path defensible. Non-operative management of an actively bleeding epidural haematoma is tenable only if reliable haemostasis can be guaranteed not for hours but across the entire reabsorption process, which here extended over weeks. The predictable, sustained FVIII activity obtained with one infusion every 48 hours - and later weekly - is what underwrote that guarantee, and the uneventful radiological course, with a single minimal increase at 24 hours followed by progressive resolution, suggests the strategy was sound. In a treatment-naïve patient with no experience of self-infusion, sustaining equivalent confidence with a standard-half-life concentrate requiring two to three daily infusions would, in our view, have been considerably harder.

These same properties extend to major surgery. In the pooled XTEND-1 and XTEND-Kids perioperative analysis, most major surgeries were managed with a single preoperative dose of 50 IU/kg - occasionally none - with a median total consumption of 163 IU/kg (days -1 to 14), substantially lower than the 489-629 IU/kg reported for an extended-half-life comparator, with excellent haemostasis, no transfusions, and no inhibitors [4]. Sustained FVIII activity allowed fewer injections, less frequent perioperative monitoring, and earlier discharge [4]. For a patient with mild HA facing major surgery, this translates directly into fewer infusions, less monitoring, and avoidance of central venous access - the same advantages we observed in a traumatic life-threatening bleed.

These clinical benefits carry an economic dimension, although its direction depends critically on the healthcare system and local pricing. In our internal pharmacoeconomic modelling of this episode, the higher per-IU acquisition cost of efanesoctocog alfa was partly offset by much lower unit consumption in the acute phase and by avoidance of both a central venous catheter and repeated infusions (9 vs. 33 administrations over the same acute period in a standard extended-half-life simulation). The overall balance, however, was highly sensitive to the duration and intensity of subsequent prophylaxis, so total acquisition cost could be higher or lower than a comparator depending on assumptions. We would therefore caution against presenting efanesoctocog alfa primarily as a cost-saving option; its value here is chiefly clinical and organisational, with efficiency accruing through fewer infusions, fewer hospital attendances, and avoidance of vascular access - components whose monetary weight varies between systems [10].

Our report has clear limitations. It is a single observation and cannot establish comparative efficacy, safety, or cost-effectiveness. The pharmacoeconomic estimate is an exploratory single-centre simulation whose direction depends on the comparator, on local pricing, and on the assumed duration of prophylaxis. Finally, the follow-up CT studies were performed at the referring neurosurgical centre and were not available to us for publication, so the radiological course is reported quantitatively in the text rather than as serial images.

## Conclusions

This case shows that efanesoctocog alfa can control a life-threatening intracranial bleed in mild HA, allowing a regimen that was progressively spaced to conventional weekly dosing, achieved without central venous access and with a low number of infusions. Two lessons have practical value beyond this single patient. First, the mild phenotype should not be equated with low risk: trauma or surgery can turn it into a haemostatic emergency, and an ultra-long-acting agent offers a pragmatic way to sustain protection in patients who are treatment-naïve and untrained in self-infusion. Second, correct laboratory monitoring is essential, since the chromogenic result must be adjusted per the manufacturer's recommendation to avoid overestimating activity and over-spacing doses. The perioperative evidence suggests that these same advantages - fewer infusions, less monitoring, and avoidance of vascular access - extend to major surgery. These observations are hypothesis-generating rather than confirmatory: a single case cannot establish comparative efficacy or cost-effectiveness, and we make no claim of generalisability; structured registries and prospective, multicentre data collection in non-severe HA are needed to aggregate what must otherwise remain individual experience, to confirm the clinical benefit and to clarify the health-economic balance, which remains strongly dependent on the healthcare system.
